# Overexpression of *TaCOMT* Improves Melatonin Production and Enhances Drought Tolerance in Transgenic *Arabidopsis*

**DOI:** 10.3390/ijms20030652

**Published:** 2019-02-02

**Authors:** Wen-Jing Yang, Yong-Tao Du, Yong-Bin Zhou, Jun Chen, Zhao-Shi Xu, You-Zhi Ma, Ming Chen, Dong-Hong Min

**Affiliations:** 1 College of Agronomy, Northwest A&F University/State Key Laboratory of Crop Stress Biology for Arid Areas, Yangling 712100, China; ywj103628@163.com; 2 Institute of Crop Sciences, Chinese Academy of Agricultural Sciences (CAAS)/National Key Facility for Crop Gene Resources and Genetic Improvement, Key Laboratory of Biology and Genetic Improvement of Triticeae Crops, Ministry of Agriculture, Beijing 100081, China; duyongtao1994@126.com (Y.-T.D.); zhouyongbin@caaas.cn (Y.-B.Z.); chenjun01@caas.cn (J.C.); xuzhaoshi@caas.cn (Z.-S.X.)

**Keywords:** wheat, melatonin, caffeic acid 3-O-methyltransferase, drought tolerance, signaling pathway

## Abstract

Melatonin (N-acetyl-5-methoxytryptamine) is involved in many developmental processes and responses to various abiotic stresses in plants. Most of the studies on melatonin focus on its functions and physiological responses in plants, while its regulation mechanism remains unknown. Caffeic acid 3-O-methyltransferase (COMT) functions at a key step of the biosynthesis process of melatonin. In this study, a COMT-like gene, *TaCOMT* (Traes_1AL_D9035D5E0.1) was identified in common wheat (*Triticum aestivum* L.). Transient transformation in wheat protoplasts determined that TaCOMT is localized in cytoplasm. *TaCOMT* in wheat was induced by drought stress, gibberellin (GA)3 and 3-Indoleacetic acid (IAA), but not by ABA. In *TaCOMT* transgenic *Arabidopsis*, melatonin contents were higher than that in wild type (WT) plants. Under D-Mannitol treatment, the fresh weight of the transgenic *Arabidopsis* was significantly higher than WT, and transgenic lines had a stronger root system compared to WT. Drought tolerance assays in pots showed that the survival rate of *TaCOMT*-overexpression lines was significantly higher than that of WT lines. this phenotype was similar to that the WT lines treated with melatonin under drought condition. In addition, the *TaCOMT* transgenic lines had higher proline content and lower malondialdehyde (MDA) content compared to WT lines after drought treatment. These results indicated that overexpression of the wheat *TaCOMT* gene enhances drought tolerance and increases the content of melatonin in transgenic *Arabidopsis*. It could be one of the potential genes for agricultural applications.

## 1. Introduction

Melatonin is a molecule with pleotropic effect in plants [1]. Since melatonin was first reported in plants in 1995, many functions of melatonin have been revealed [2,3]. At present, most of the studies on melatonin focus on biosynthetic pathways and its multifunctional roles in plants. For the melatonin biosynthetic pathway in plants, tryptophan is decarboxylated and translated into tryptamine, then tryptamine is further hydroxylated and translated into serotonin. Thereafter, serotonin is converted to N-acetyl serotonin and catalyzed by serotonin N-acetyltransferase (SNAT), and N-acetyl serotonin is then methylated by acetyl serotonin methyl transferase (ASMT) resulting in the formation of melatonin [4,5]. Excepting ASMT, the caffeic acid O-methyltransferase (COMT) can also catalyze N-acetyl serotonin into melatonin in *Arabidopsis* [6,7]. In rice, the biosynthesis of melatonin also requires the N-acetylserotonin methyltransferase activity of COMT [8]. 

Melatonin plays important roles in regulating plant growth and development and enhancing the resistance of plants against biotic and abiotic stresses [9,10,11,12]. For example, melatonin treatment significantly enhances the drought tolerance of wheat seedlings with the decreasing of membrane damage, increasing photosynthetic rate, maintaining intact grana lamella of chloroplast, and increasing water holding capacity [13]. Melatonin systemically reduced drought stress-induced damage in *Medicago sativa* plants by modulating nitro-oxidative homeostasis and proline metabolism [9]. Overexpression of the apple *MzASMT* gene improves the production of melatonin and enhances resistance to drought in *Arabidopsis* [14]. Overexpression of *TaCOMT-3D* improves wheat resistance to sharp eyespot disease and promotes lignin accumulation in stems of wheat [15]. Melatonin-induced *CBF/DREB1s* are essential for diurnal changes in disease resistance in *Arabidopsis* [16]. Although multiple studies have shown that melatonin is important in many biological processes, regulation mechanisms of melatonin in plants are not known [5,10,17]. 

Wheat is an important food crop in the world and it is mainly grown in arid or semi-arid regions. Similar to the other gramineous crops, drought is one of the main limiting factors affecting wheat growth and yield [18,19]. With the completion of wheat genome sequencing, more and more stresses associated genes have been reported in wheat. In this study, we identified a wheat COMT-like gene, *TaCOMT*, and determined that *TaCOMT* is induced by drought and gibberellin (GA) in wheat. Overexpression of *TaCOMT* promoted the synthesis of melatonin and enhanced drought tolerance of transgenic lines, which is a novel abiotic stress regulation mechanism in *Arabidopsis*. 

## 2. Results

### 2.1. Conserved Domain Analysis of TaCOMT, Expression Profile under Group Stresses and Phytohormones, and Subcellular Localization of TaCOMT in Wheat

**Figure 1:**
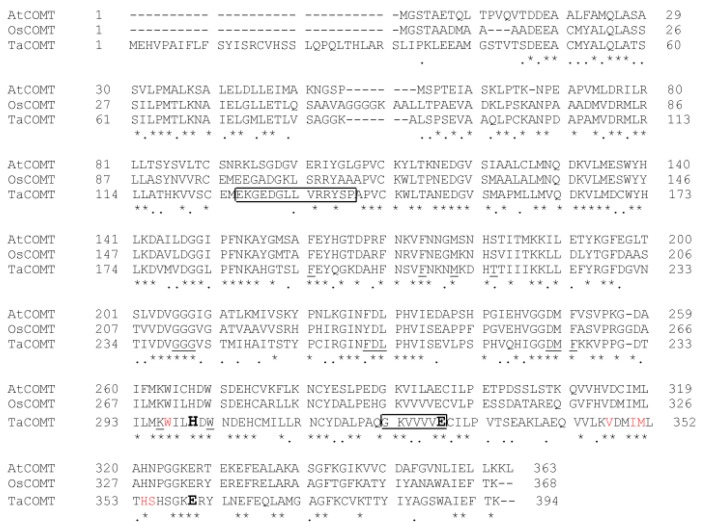
Multiple alignments of TaCOMT. Multiple sequence alignments were constructed with DNAMAN. The three completely identical catalytic residues (His267, Glu295, and Glu327) of caffeic acid 3-O-methyltransferases (COMTs) are shown in bold letters. The SAM-binding sites are underlined. The phenolic substrate binding sites are in red letters. The putative N-acetylserotonin (NAS) binding domains are in boxes. The same amino acids are marked with asterisks (*).

*TaCOMT* has an open reading frame of 1185 bp which encodes a protein with 395 amino acids and a molecular weight was 43.67 KD. The isoelectric point of TaCOMT was approximately 6.05. To find the conserved domain of the TaCOMT protein, the amino acid sequence of *Arabidopsis* AtCOMT and rice OsCOMT were used for multiple sequence alignment, and results showed that TaCOMT exhibited 54.19% and 60.2% sequence identity with AtCOMT and OsCOMT, respectively. Although the homology between different plant species is relatively low, those COMT-like protein have some conserved sites including N-acetylserotonin (NAS) binding domains [6,8], catalytic sites, S-adenosyl-L-methionine (SAM)-binding sites, and phenolic substrate binding sites, which are very important for COMT-like protein functions (Figure 1). The putative N-acetylserotonin (NAS) binding domains, catalytic residues (His267, Glu295, and Glu327), SAM-binding sites, and the phenolic substrate binding sites are shown in Figure 1. 

**Figure 2:**
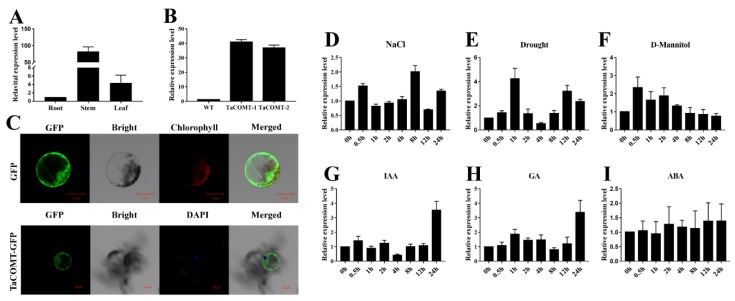
Tissue expression specificity, expression patterns under group stresses and phytohormones and subcellular localization of TaCOMT. Analysis of transcriptional level of *TaCOMT* in roots, stems, and leaves by qRT-PCR (**A**). Expression of *TaActin* as a loading control. Expression analysis of transgenic lines by qRT-PCR (**B**). Expression of *AtActin* as a loading Control. Expression patterns of *TaCOMT* after treatment with NaCl (**D**), Drought (**E**), D-Mannitol (**F**), 3-Indoleacetic acid (IAA) (**G**), gibberellin (GA) (**H**), and ABA (**I**) for 0, 0.5, 1, 2, 4, 8, 12 and 24 h. Analysis of transcriptional level by qRT-PCR assay. Expression of *TaActin* as a loading control. The data are shown as the means ± SDs of three experiments. Transient expression of 35S::TaCOMT-GTP with DAPI staining and GFP control vectors in wheat protoplasts (**C**). Scale bars = 20 μm.

Tissue specificity expression analysis showed that the expression level of *TaCOMT* in the wheat stem was 54.7 times higher than in wheat root and 10.5 times higher than in wheat leaf (Figure 2A). The *TaCOMT* gene was induced by stresses and phytohormones such as drought (Figure 2E), D-Mannitol (Figure 2F), 3-Indoleacetic acid (IAA) (Figure 2G), GA (Figure 2H), and ABA (Figure 2I), and the highest expression peaks were 4.1, 3.2, 3, 3.1, and 1.2 times higher, respectively, than those before treatment controls (Figure 2). In addition, transcription of *TaCOMT* was slightly upregulated by salt and reached a maximum level at 8 h (A) (Figure 2D). As shown in the Figure 2C, subcellular localization analysis revealed that TaCOMT may localize in the cytoplasm.

### 2.2. TaCOMT Conferred Drought Tolerance in Transgenic Arabidopsis

**Figure 3:**
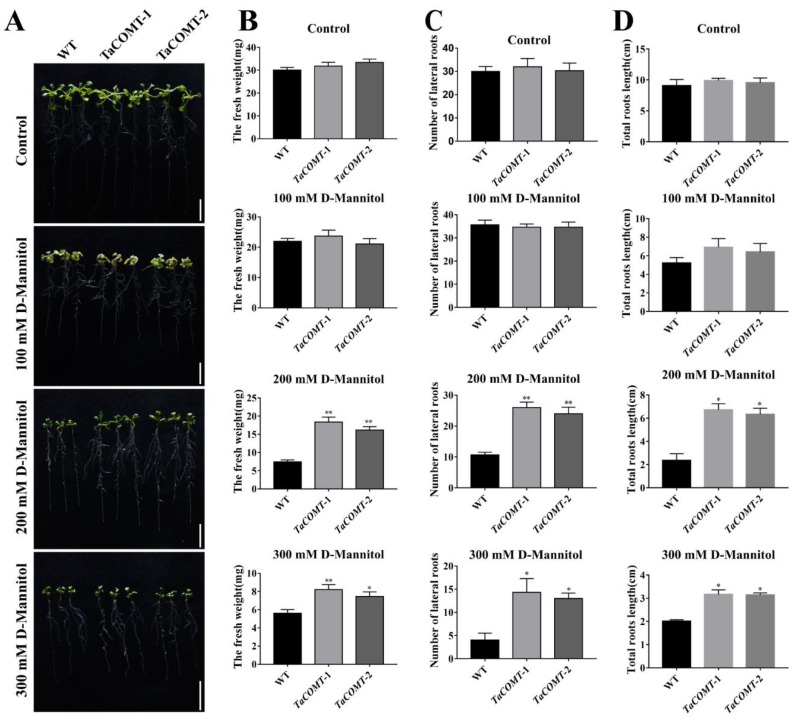
The phenotype of *TaCOMT* transgenic lines under D-Mannitol treatment. Six-day-old seedlings of WT and *TaCOMT* transgenic lines were transferred to ½ MS medium containing 0, 100, 200, or 300 mM D-Mannitol. A week later, the phenotype of roots was investigated (bar = 2 cm) (**A**). The statistical results of fresh weight (**B**), number of lateral roots (**C**), and total root length (**D**) of wild type (WT) and transgenic lines are shown in Figure 5. At least 25 seedlings per phenotype were counted. The data are shown as the means ± SDs (*n* = 25) of three experiments. ANOVA test identified significant differences (* *p* < 0.05, ** *p* < 0.01).

After transformation of *Arabidopsis*, we obtained two independent T_3_ generation *TaCOMT*-overexpression lines. The expression of wheat *TaCOMT* gene in transgenic lines (*TaCOMT*-1 and *TaCOMT*-2) were higher 40 and 38 times than that of WT lines (Figure 2B). In order to verify drought resistance of transgenic *Arabidopsis*, D-mannitol was used to simulate drought treatment in medium. The results of drought tolerance assay showed that under 0 mM D-mannitol and 100 mM D-mannitol treatments, there was no significant difference in plant fresh weight and root phenotypes, including branch number of lateral roots and total root length (Figure 3). Under 200 mM D-mannitol treatment, the plant fresh weight of transgenic lines *TaCOMT*-1 and *TaCOMT*-2 were 18.36 mg and 16.17 mg, respectively, significantly higher than that of WT (7.43 mg) (*p* < 0.01) (Figure 3B). The branch number of lateral roots in transgenic lines *TaCOMT*-1 and *TaCOMT*-2 were 26 and 24, respectively, significantly higher than that of WT (10.67) (*p* < 0.01) (Figure 3C). The total root length of transgenic lines *TaCOMT*-1 and *TaCOMT*-2 were 6.72 cm and 6.33 cm, respectively, also significantly higher than that of WT (2.35 cm) (*p* < 0.05) (Figure 3D). Under 300 mM D-Mannitol treatment, phenotype differences between transgenic plants and WT were similar (Figure 3).

**Figure 4:**
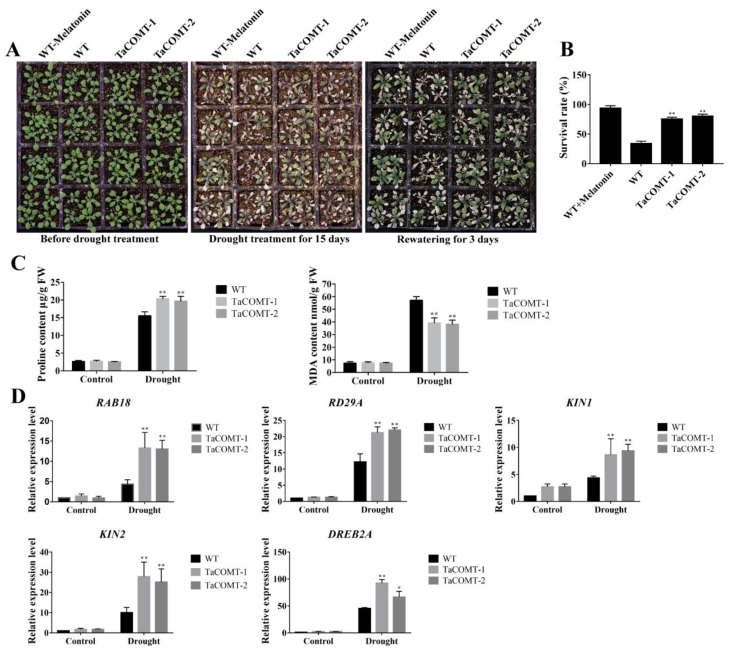
*TaCOMT* improved drought tolerance in transgenic plants. Three-week-old seedlings were treated without irrigation for 15 days (**A**). After three days of rehydration, the survival rates of WT and transgenic plants were counted (**B**). The contents of proline and malondialdehyde (MDA) of *Arabidopsis* seedlings treated with drought for 10 days (**C**). The two-week-old *Arabidopsis* seedlings were placed on filter paper to simulate drought for two hours, and then the transcription level of drought-responsive genes was analyzed (**D**). Expression of *AtActin* as a loading control. At least 25 seedlings per phenotype were counted. The data were shown as the means ± SDs (*n* = 25) of three experiments. An ANOVA test identified significant differences (* *p* < 0.05, ** *p* < 0.01).

For drought tolerance assay, the three-week-old seedlings were grown for 15 days without irrigation in the culture chamber, and then re-watered for 3 days. The survival rates of transgenic lines *TaCOMT*-1 and *TaCOMT*-2 (75% and 80%, respectively) were significantly higher than that of WT (33.75%) (Figure 4A,B). WT plants were sprayed with 10 µΜ melatonin (twice each week) after drought treatment for 15 days. The survival rate of WT with melatonin treatment was 93.75%, which was similar to transgenic *Arabidopsis* and significantly higher than that of WT without melatonin treatment (Figure 4A,B). These results suggested that overexpression of wheat *TaCOMT* gene enhanced drought tolerance of transgenic *Arabidopsis*, which may be related to melatonin level in plants. 

**Figure 5:**
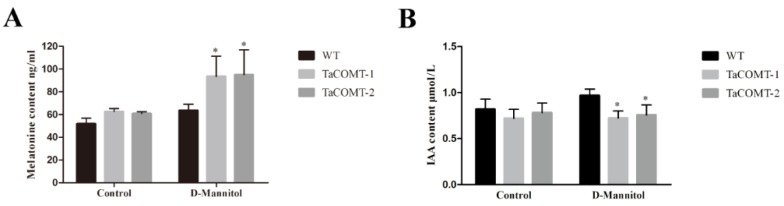
The contents of melatonin and IAA. Six-day-old Arabidopsis seedlings growth on ½ MS medium were treated with 200 mM mannitol for a week. Then, 0.1 g seedlings samples were used to detect the contents of melatonin (**A**) and IAA (**B**). Normal growth of Arabidopsis seedlings as a control. The data are shown as the means ± SDs of three experiments. An ANOVA test detected significant differences (* *p* < 0.05, ** *p* < 0.01).

In order to explore the potential physiological mechanism of *TaCOMT*-overexpression lines to improve drought tolerance, we determined the contents of proline and MDA in *TaCOMT*-overexpression plants and WT plants under normal and drought growth conditions. Under normal growth condition, the contents of proline and MDA in transgenic and WT plants were similar (Figure 4C). Under drought conditions, the accumulation of proline in transgenic lines (*TaCOMT*-1 and *TaCOMT*-2) were 20.31 and 19.55 µg/g, respectively, which were significantly higher than that of WT (15.51 µg/g) (Figure 4C). The content of MDA in *TaCOMT*-overexpression lines were 39.15 and 38.25 nM/g, respectively, which were significantly lower than that of WT (56.98 nM/g) (Figure 4C). These results suggested that overexpression of *TaCOMT* maintains intracellular osmotic balance and reduces cell membrane damage, which contributed to drought tolerance of transgenic *Arabidopsis*. We also analyzed the melatonin content in WT and transgenic lines. The results showed that, under normal conditions, the melatonin content of transgenic lines *TaCOMT*-1 and *TaCOMT*-2 were 62.63 and 60.84 ng/mL, respectively, which were slightly higher than the 51.88 ng/mL in WT (Figure 5A). Under D-Mannitol treatment, the melatonin content of transgenic *TaCOMT*-1 and *TaCOMT*-2 were 86.84 and 95.01 ng/mL, respectively, which were significantly higher than the 60.54 ng/mL in WT (*p* < 0.05) (Figure 5A). These data indicating that overexpression of *TaCOMT* increased biosynthesis of melatonin in transgenic *Arabidopsis* under drought stress. In addition, we detected the content of IAA in WT and transgenic lines under normal and D-Mannitol treatment. Under normal conditions, the content of IAA of transgenic lines *TaCOMT*-1 and *TaCOMT*-2 were 0.72 and 0.77 µmol/L, respectively, which were slightly lower than the 0.81 µmol/L in WT. Under D-Mannitol treatment, the IAA content of transgenic lines (*TaCOMT*-1 and *TaCOMT*-2) were 0.70 and 0.73 µmol/L, respectively, which was significantly lower than that of WT lines (0.65 µmol/L) (*p* < 0.01) (Figure 5B).

### 2.3. Some Stress-Responsive Genes Were Induced in TaCOMT Transgenic Arabidopsis

So as to identify downstream genes of *TaCOMT* in transgenic *Arabidopsis*, the expression of some stress responsive genes [20,21,22] that involved in drought pathway were analyzed. qRT-PCR revealed that expression of some genes such as *AtCOR15A* [23], *AtCOR47* [24], and *AtP5CS1* [25] was not different between transgenic *Arabidopsis* and WT lines under normal and drought conditions (Figure S1). However, under drought conditions the expression of some genes including *AtRD29A* [26], *AtRAB18* [27], *AtKIN1* [28], *AtKIN2* [29], and *AtDREB2A* [30] in transgenic *Arabidopsis* were significantly higher than that of WT (Figure 4D). For example, the expression of *AtDREB2A* in transgenic plants was 92.58 and 66.33 times higher than that of WT under drought condition, whereas its expression was not significantly different compared to WT under normal growth conditions. These results indicated that overexpression of *TaCOMT* enhances drought resistance in transgenic *Arabidopsis* by regulating transcription of downstream drought-responsive genes.

### 2.4. TaCOMT Improved Drought Tolerance through an ABA-Independent Pathway in Arabidopsis

**Figure 6:**
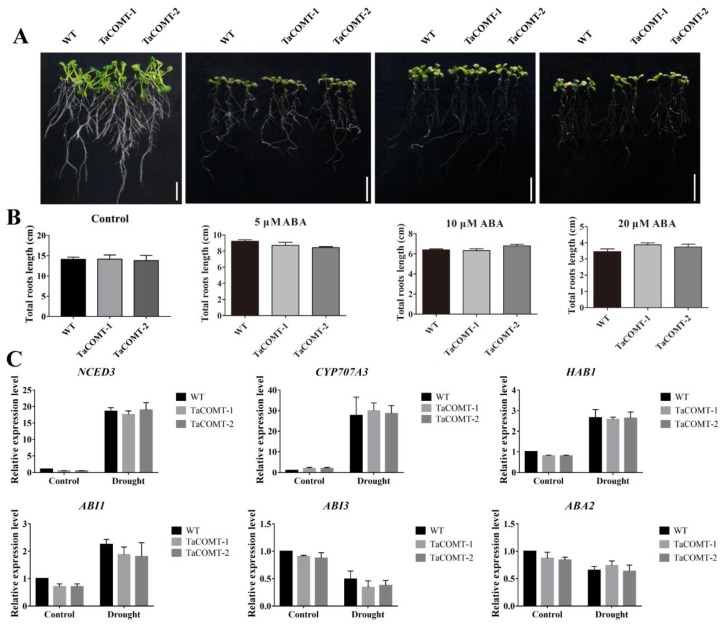
The root length of ABA treatment and expression level of ABA-responsive genes in transgenic lines. The six-day-old seedlings of WT and *TaCOMT* transgenic lines were transferred to ½ MS medium containing 0, 5, 10 and 20 µM ABA. A week later, the phenotype of roots was investigated (bar = 2 cm) (**A**). The total root length of WT and transgenic lines were statistically analysis (**B**) For the expression level of ABA-responsive genes, two-week-old Arabidopsis seedlings were placed on filter paper for two hours to simulate drought, and then the transcription level of ABA-responsive genes was analyzed (**C**). Expression of *AtActin* as a loading control. At least 25 seedlings per phenotype were counted. The data are shown as the means ± SDs (*n* = 25) of three experiments. ANOVA test demonstrated significant differences (* *p* < 0.05, ** *p* < 0.01).

ABA plays important roles in the drought response process in plants [31]. In order to investigate whether the *TaCOMT* transgenic lines responded to ABA treatment, six-day-old seedlings were treated with various concentrations of ABA for 7 days. In the absence of ABA, there were no differences in root growth between *TaCOMT*-overexpression and WT lines. With ABA treatment, root growth was inhibited both in transgenic *Arabidopsis* and WT lines, whereas phenotypes were no different between *TaCOMT*-overexpression and WT lines (Figure 6A). The statistical analysis of total root length showed that there was no difference between transgenic *Arabidopsis* and WT lines under treatment with different ABA concentrations (Figure 6B). In addition, we investigated the expression of genes involved in the ABA pathway including ABA synthesis, metabolism, and signal transduction, such as *AtNCED3* [32], *AtCYP707A3* [33], *AtHAB1* [34], *AtABA3* [35], and *AtABI1* [36,37] under drought treatment. As shown in Figure 6C, the transcription level of all of the above genes shows that they were induced by drought treatment, but the expression of the ABA-related genes showed no difference between *TaCOMT*-overexpression and WT lines under normal and drought stress, which indicates that enhanced drought tolerance in *TaCOMT*-overexpression lines was independent of the ABA pathway. In addition, we also analyzed heat stress tolerance of *TaCOMT*-overexpression lines, and the results showed that there was no difference between transgenic and WT lines under heat stress (Figure S2). 

### 2.5. TaCOMT Enhanced the Response of Transgenic Plants to GA3 by Regulating Expression of GA Metabolism-Related Genes

**Figure 7:**
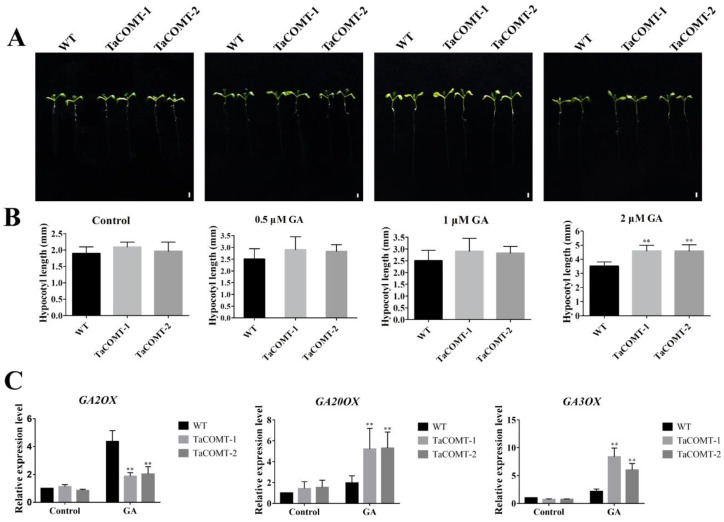
Hypocotyl length and expression of GA-associated genes under GA treatment in *Arabidopsis*. Phenotypes of WT and *TaCOMT* transgenic *Arabidopsis* seeds growth on medium containing 0, 0.5, 1 and 2 µΜ GA for a week (bar = 2 mm) (**A**). Statistical results of hypocotyl length of WT and transgenic plants treated with different concentrations of GA (**B**). Two-week-old Arabidopsis seedlings were transferred to ½ MS medium containing 100 µM GA for 24 h, and then the transcription level of GA-responsive genes was analyzed (**C**). Expression of *AtActin* as a loading control. At least 25 seedlings per phenotype were counted. The data were shown as the means ± SDs (*n* = 25) of three experiments. An ANOVA test showed significant differences (* *p* < 0.05, ** *p* < 0.01).

In expression profile analysis, we found that *TaCOMT* was induced by GA3 treatment (Figure 2H). Therefore, we analyzed the response of *TaCOMT* transgenic lines under GA3 treatment. under normal and 0.5 µΜ GA3 treatment conditions, the length of the hypocotyl was no difference between *TaCOMT*-overexpression and WT lines (Figure 7A). Hypocotyl length varied with different concentrations of GA3 hormone (Figure 7B). Under 1 µΜ GA3 treatment, the hypocotyl of transgenic lines *TaCOMT*-1 and *TaCOMT*-2 were 4.69 and 4.47 cm, respectively, which were significantly longer than 3.64 cm in WT (*p* < 0.01) (Figure 7B). The situation under 2 µΜ GA3 treatment was similar to the 10 µΜ GA3 treatment: the hypocotyl of transgenic lines *TaCOMT*-1 and *TaCOMT*-2 were 4.59 and 4.56 cm, respectively, which were significantly longer than that of WT at 3.51 cm (*p* < 0.01) (Figure 7B). We examined the expression levels of GA-related genes, including GA synthesis inhibition-related gene *GA2OX* [38] and GA synthesis-related genes *GA20OX* [39] and *GA3OX* [40]. We found that the expression of *GA20OX* and *GA3OX* genes in transgenic lines were significantly higher than that of WT, and the expression of *GA2OX* was significantly lower than that of WT (*p* < 0.01) (Figure 7C). Additionally, we found that under 24 h dark cycles, there was no difference in hypocotyl between transgenic and WT plants treated with or without paclobutrazol (PAC), which is a GA inhibitor (Figure S3). Those results may indicate that *TaCOMT* is involved in the GA pathway by regulating the expression of GA-associated genes.

Moreover, we found other phenotype changes between *TaCOMT*-overexpressing and WT lines. There was no difference between transgenic and WT lines after three weeks of growth. However, six days later, we observed that transgenic *Arabidopsis* showed an earlier flowering phenotype than WT *Arabidopsis* (Figure S4A). The number of rosette leaves in *TaCOMT*-overexpressing lines were less than that of WT (Figure S4B). In addition, we investigated the expression levels of some flowering-related genes, *FT* [41] and *SOC1* [42], and found that the expression levels of *FT* and *SOC1* in transgenic lines were significantly higher than those in the WT (Figure S4C). This suggested that *TaCOMT* could promotes early flowering of transgenic plants by regulating the transcription of genes related to flowering.

## 3. Discussion

COMT is an O-methyltransferase that can potentially act in various branches of the phenylpropanoid pathway. A wheat caffeic acid 3-O-methyltransferase *TaCOMT-3D* positively contributes to both resistance to sharp eyespot disease and stem mechanical strength by promoting lignin synthesis [15]. In this study, melatonin content was increased in *TaCOMT*-overexpression lines, and *TaCOMT*-overexpression lines had stronger drought tolerance as compared to the WT lines (Figure 3, Figure 4 and Figure 5). Moreover, spraying of melatonin also enhanced tolerance of WT plants, which were similar to the response of *TaCOMT*-overexpression lines without melatonin treatment (Figure 4A). Those results indicate that *TaCOMT* could confer drought tolerance to plants by increasing melatonin biosynthesis. In addition, the *TaCOMT* transgenic lines were no different compared to WT lines under ABA treatment (Figure 6A,B) and under drought treatment, the expression of ABA-related genes was no difference between WT and *TaCOMT*-overexpression lines. (Figure 6C). Those results suggested that enhanced drought tolerance of transgenic lines was not related with ABA pathway. 

Plant hormones are important regulatory factors in plant growth and development. Gibberellin (GA) is one of six plant hormones involved in many development processes of plants, such as seed germination, stem elongation, and development of flower, seed, and fruit [43,44]. Previous studies have shown that melatonin alleviates ROS damage and enhances ABA degradation while promoting GA biosynthesis during seed germination of cucumber. In this study, we found that expression of *TaCOMT* was induced by GA (Figure 7). Under GA treatment, the hypocotyl of transgenic *Arabidopsis* lines was significantly longer than that of WT (Figure 7B), suggesting that *TaCOMT* is involved in the GA pathway. Overexpression of *TaCOMT*, however, promoted early flowering of *TaCOMT* transgenic *Arabidopsis* compared to WT (Figure S4A) and the expression level of flower-associated genes were significantly higher than that of WT (Figure S4C). Previous reports suggest that increasing GA content will promote early flowering in plants. In our study, overexpression of *TaCOMT* enhanced *AtGA20ox* and *AtGA3ox* gene expression (Figure 7C), similar to the results of Zhang et al. [44]. Those results suggested that *TaCOMT* may promote early flowering by synthesizing melatonin, leading to increased GA content.

Auxin regulates a variety of cellular and developmental responses in plants, including cell division, expansion, and differentiation, and the distribution of growth between primary and lateral roots and stem meristem [45]. Wand et al. observed that the Micro-Tom tomato transgenic plants overexpressing the sheep *oAANAT* and *oHIOMT* genes responsible for the last two steps of melatonin synthesis were more branching than the controls due to regulation of IAA content [5]. In our study, overexpression of *TaCOMT* in *Arabidopsis* increased the level of melatonin and decreased the content of IAA compared with WT plants (Figure 5). Under drought conditions, the difference between transgenic lines and WT lines were significantly (Figure 4A), but we did not find that *TaCOMT* transgenic lines had more branching than WT *Arabidopsis*, which may require long-term observation or further study in wheat. The relationship between auxin and gibberellin is complex [46] and Ross et al. showed that a normal level of auxin is necessary to maintain the biological activity of gibberellin [47]. We found that *TaCOMT* may involve in GA signal pathway and reduced the content of IAA in *TaCOMT*-overexpression lines (Figure 7 and Figure 5B). This suggests that melatonin may affect GA synthesis or that there is an interaction between melatonin and GA at a specific time, which in turn affects plant growth. The mechanism needs further study. Zuo et al. reported that IAA and melatonin have a substrate competition relationship by sharing the same precursor, tryptophan [14]. We found that overexpression of *TaCOMT* reduced the content of IAA, which supports the mechanism in synthesis of melatonin by the competitive binding of substrate tryptophan. 

**Figure 8:**
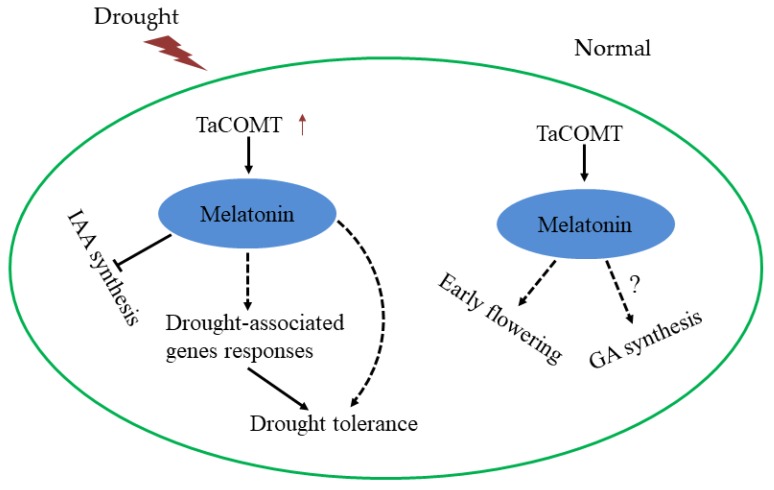
Proposed mechanisms of overexpression of *TaCOMT* to enhance drought tolerance and participate in GA and IAA Pathway in *Arabidopsis.* Under drought condition, the expression of *TaCOMT* increased melatonin synthesis in plants. The increasing of melatonin content leads to the decrease of IAA level and the changing of transcription of drought-related genes, and, then, enhancing drought tolerance in plants. The increasing of melatonin content can also improve the drought tolerance of plants. Under normal conditions, overexpression of *TaCOMT* increased the content of melatonin in plants. High levels of melatonin promoted early flowering and involved in the GA pathway.

According to the above results, a model of how *TaCOMT* enhances drought tolerance of transgenic *Arabidopsis* and participates in GA and IAA signaling pathway was suggested (Figure 8). In the future, this gene may be one of the candidate genes for wheat resistance breeding. Wheat is one of the main food crops in the world. However, abiotic stress has seriously affected wheat yield and quality. Previous studies found that melatonin is a potential scavenger of reactive oxygen species (ROS) and reactive nitrogen species (RNS) in plants. and with increased content of melatonin, it could improve plant tolerance to abiotic stress [13,48]. Melatonin affects plant development by integrating various factors including plant endogenous hormone content, enzymes, and various signaling molecules to affect lateral root growth, circadian rhythm, flowering time, and biomass yield in plants. [14,48,49,50]. Recently, increasing production of melatonin through genetic engineering has been shown to be feasible. COMT encodes a caffeic acid 3-O-methyltransferase that catalyzes the synthesis of melatonin from tryptophan substrates and improves melatonin content in plants, which improves plant resistance and promotes plant development. This research and the application of COMT genes is thus of great significance for wheat molecular breeding. 

## 4. Materials and Methods

### 4.1. Isolation and Bio-Informative Analysis of the TaCOMT Gene

The full-length cDNA of *TaCOMT* was downloaded from GenBank database (https://www.ncbi.nlm.nih.gov/genbank/). The coding sequence was amplified using the pEASY^®^-Uni Seamless Cloning and Assembly Kit with gene specific primers (F1: 5′-AGATGAATGGGTGGATTCGTGTG-3′, R1: 5′-CTCCAGAAAATGATCCAAGTAAAAT-3′, and F2: 5′-GTGTGTGTTACAAATGGTTTAGTGA-3′, R2: 5′-CTTAGGAACACCGAGAATACGATGC-3′); the specific primers were designed by Primer Premier 5.0 software.

To find the conserved domain of the TaCOMT amino acid sequence, the sequences of AtCOMT and OsCOMT (Uniprot accession number: Q9FK25 and Q6ZD89) were downloaded from Uniprot (https://www.uniprot.org/). The theoretical iso-electric point and molecular weight was predicted using pI/Mw tool (http://web.expasy.org/compute_pi/). DNAMAN software was used to analyze sequence identity. 

### 4.2. Plant Materials and Stress Treatments 

Wheat seeds (*T. aestivum* L. cultivar Xiaobaimai) were grown in Hoagland liquid medium for 10 days at 22 °C with 16 h light/8 h dark photoperiod (100 μΜ m^− 2^ s^− 2^). For drought treatment, the wheat seedlings were placed on filter paper to simulate drought for 0, 0.5, 1, 2, 4, 8, 12 and 24 h. For salt, GA, IAA, and ABA treatments, seedling roots were immersed in half-strength Hoagland solution containing 100 mM NaCl, 100 µM GA3, 10 µM IAA, or 100 µM ABA and sampled at 0, 0.5, 1, 2, 5, 10, 24, and 48 h. The samples were then dropped immediately into liquid nitrogen and stored at −80 °C for RNA extraction.

### 4.3. Generation of Transgenic Arabidopsis

The complete open reading frame (ORF) of the *TaCOMT* gene was cloned into pCAMBIA1302 driven by the cauliflower mosaic virus (CMV) 35S promoter. The recombinant plasmids were confirmed by sequencing and then introduced into *Agrobacterium tumefaciens* strain GV3101 (Biomed, Beijing, China) using the freeze–thaw method [7,51]. The vector of pCAMBIA1302-*TaCOMT* was transformed into WT (Col-0) *Arabidopsis* plants using the floral-dip method [52]. The seeds of transformed plants were cultured on MS medium (Duchefa, Haarlem, Holland) with 50 mg/L hygromycin (Sigma, Louis, USA) for 10 days to obtain positive plants with T-DNA insertion. These plants were transferred to soil until the T_3_ generation.

For further experiments, we detected the expression level of *TaCOMT* in *Arabidopsis thaliana*. The *AtActin* gene was used as an internal control. Each experiment included three replicates.

### 4.4. Drought and ABA Assay in Arabidopsis

Surface-sterilized seeds of WT and transgenic lines were sown on ½ MS medium (Duchefa) and subjected to low temperature treatment at 4 °C for 3 days to synchronize germination. The seeds were transferred to a growth chamber at 22 °C and 70% relative humidity with a 24 h light/dark cycle (16:8 light/dark).

For root growth assay, six-old-day seedlings were transferred to ½ MS medium with different concentrations of D-Mannitol (0, 100, 200, and 300 mM) (Merck, Kenilworth, USA) and ABA (0, 5, 10, and 20 µM) (Sigma, USA). Photos were taken after 7 days and the total root length was evaluated using an Epson Expression 11000XL root system scanning analyzer (Epson, Nsgano Prefecture, Japan) [53]. At least 25 seedlings per genotype were measured.

For drought treatment in later stages, three-week-old seedlings were treated without irrigation for 15 days. Phenotype pictures were taken, and the survival rate was counted after re-watering for 3 days. The survival rate was calculated by dividing the number of plants alive after rehydration for 3 days by the total number of plants surveyed. At least 25 seedlings from each line were measured. Each experiment included three replicates.

### 4.5. Heat Assay in Arabidopsis 

Surface-sterilized seeds of WT and transgenic lines were transferred to a growth chamber (22 °C) after being subjected to low temperature treatment at 4 °C for 3 days. For heat assays, the six-day-old seedlings were subjected to 37 °C for an hour and then recovered for 2 h at normal conditions. Then after treatment at 44 °C for 4.5 h, the seedlings were transferred to normal conditions for 5 days. Each experiment included three replicates. 

### 4.6. RNA Extraction and Gene Expression Analysis

Total RNA was extracted from leaves of transgenic and WT plants according to the manufacturer’s protocol of plant total RNA kit (ZOMANBIO, ZP405-1, Beijing, China). RNA integrity was analyzed by spectrophotometry and 1% agarose gel electrophoresis. 1μg RNA was took for cDNA synthesis using TransScript One-Step gDNA Removal and cDNA Synthesis SuperMix kit (TransGen, Beijing, China) following the manufacturer’s protocol. Real-time RT-PCR analyses were performed using TransStart Top Green qPCR SuperMix (+Dye II) (TransGen, Beijing, China). The three-step method was performed as follows: 94 °C 30 s; 40 cycles of 94 °C for 5 s, 58 °C for 15 s and 72 °C for 34 s. The *Actin* gene was used as an internal control. After the amplification process, the relative quantification of gene expression and statistical analysis were calculated using the 2^−ΔΔ*C*T^ [54]. All primers used for qRT-PCR are shown in Table S1.

### 4.7. Subcellular Localization of TaCOMT

To determine the subcellular localization of TaCOMT, the full-length *TaCOMT* cDNA was amplified using a pair of primers (F: 5′-TATCTCTAGAGGATCCATGGAGCATGTTCC-3′, and R: 5′-TGCTCACCATGGATCCTTTTGTAAATTCAATAG-3′) both containing the BamH I restriction site. The resulting PCR product was cloned into the vector 16318hGFP, which was driven by the CaMV 35S promoter, using an In-Fusion HD Cloning Kit (TransGen, Beijing, China). The plasmids were introduced into wheat protoplasts via the PEG4000-mediated method [55] for about 12 h. The protoplasts of TaCOMT-GFP were stained with 0.1 µg/mL DAPI for 3–5 min before observation. The fluorescence of GFP in protoplast cells was visualized using a confocal microscope (Zeiss LSM 700, CarlZeiss, Oberkochen, Germany) and images were acquired with ZEN 2010 software(CarlZeiss, Oberkochen, Germany).

### 4.8. Length of Hypocotyl under GA3 and PAC Treatment

For gibberellin (GA3) treatment, the surface-sterilized seeds of WT and transgenic lines were sown on ½ MS medium with different concentrations of GA3 (0, 0.5, 1, and 2 µM) (Solarbio, Beijing, China), and subjected to low temperature treatment at 4 °C for 3 days to synchronize germination. The seeds were transferred to a growth chamber at 22 °C and 70% relative humidity with a 24 h light/dark cycle (16:8 light/dark). For PAC treatment, similarly, the seeds of WT and transgenic lines were sown on ½ MS medium with different concentrations of PAC (0, 1, 10, and 20 nM) (Solarbio, Beijing, China), and then grown at 4 °C for 3 days. The seeds were transferred to a growth chamber at 22 °C and 70% relative humidity under 24 h dark. Pictures were taken after 6 days and the length of the hypocotyl was evaluated using vernier calipers. At least 25 seedlings per phenotype were counted.

### 4.9. Melatonin and Auxin Content in Transgenic Plants

The seedlings of the transgenic and WT plants treated with 200 mM D-Mannitol for one week as described above were extracted to measure the contents of melatonin and 3-Indoleacetic acid (IAA) following the Melatonin ELISA Kit (Jianglaibio, JL-F14098, Beijing, China) and Auxin ELISA Kit (Jianglaibio, JL13982, Beijing, China) protocols. Seedlings under normal growth conditions were used as controls. In brief, 0.1 g of seedling samples were quick-frozen in liquid nitrogen and ground to fine power using a grinding machine followed by adding 0.5 mL 0.01M PBS (pH 7.4) and vortexing. For further extraction, the homogenate could be shaken or freeze-thawed repeatedly. Then, the mix was spun at 5000× *g* for 5 min and the supernatant was transferred to a fresh centrifuge tube for melatonin and IAA quantification according to the manufacturer’s instructions (Jianglaibio, Beijing, China). Each experiment included three replicates.

### 4.10. The Content of MDA and Proline in Transgenic Plants

The three-week-old seedlings of transgenic and WT plants were subjected to drought for 10 days. Then, 0.1 g of leaves was isolated from the seedlings to determine the concentration of proline and malondialdehyde (MDA) according to the manufacturer’s protocols of proline assay kit (Comin, PRO-1-Y, Suzhou, China) and MDA assay kit (Comin, MDA-1-Y, China). Each experiment included three replicates.

### 4.11. Statistical Analysis

One-way ANOVA was used for statistical analyses by GraphPad Prism 5 software (GraphPad Company, San Diego, CA, USA) [56]. *p*-value < 0.05 was considered statistically significant.

## 5. Conclusions

Caffeic acid 3-O-methyltransferase (COMT) is a key enzyme in the biosynthesis process of melatonin. The upregulation of *TaCOMT* in *Arabidopsis* positively relates to melatonin content. and enhanced plant drought resistance. Furthermore, *TaCOMT* affected GA response and flowering time of transgenic *Arabidopsis* by regulating expression of GA metabolism and flowering-related genes. These results contribute to applications of *COMT* genes in wheat breeding and increase our understanding of the regulation mechanism of the melatonin in plants. 

## Supplementary Materials

Supplementary materials can be found at http://www.mdpi.com/1422-0067/20/3/652/s1. Figure S1. The stress associated genes not response to drought in transgenic *Arabidopsis*. The two-week-old Arabidopsis seedlings were placed on filter paper to simulate drought for two hours, and then the transcription level of drought-responsive genes was analyzed (A–C). Expression of *AtActin* as a loading control. An ANOVA test identified significant differences (* *p* < 0.05, ** *p* < 0.01). Figure S2. The phenotype of *TaCOMT* transgenic lines under heat treatment. The phenotype of WT and transgenic lines were treated with 44 for 4.5 h. Figure S3. Hypocotyl length under PAC treatment in *Arabidopsis*. Phenotypes of WT and *TaCOMT* transgenic *Arabidopsis* seeds grown on medium containing 0, 1, 10, and 20 nΜ PAC for a week (A). Statistical results of hypocotyl length of WT and transgenic plants treated with different concentrations of PAC (B). At least 25 seedlings per phenotype were counted. The data are shown as the means ± SDs (*n* = 30) of three experiments. ANOVA test identified significant differences (* *p* < 0.05, ** *p* < 0.01). Figure S4. The phenotype of early flowering in *TaCOMT* transgenic lines. Three-week-old seedlings under normal conditions. After six days, *TaCOMT*-overexpression lines showed an earlier flowering phenotype than WT (A). The number of rosette leaves in flowering of transgenic and WT lines (B). The leaves of three-week-old *Arabidopsis thaliana* seedlings were used to analyze the expression of flowering-associated genes (C). Expression of *AtActin* was used as a loading control. At least 25 seedlings per phenotype were counted. The data are shown as the means ± SDs (*n* = 25) of three experiments. ANOVA test demonstrated significant differences (* *p* < 0.05, ** *p* < 0.01). Table S1. Primers used for qRT-PCR assays.

## Abbreviations

TFs	transcription factors
qRT-PCR	Quantitative real-time PCR
MDA	malonaldehyde
PEG	polyethylene glycol
WT	wild type
COMT	Caffeic acid 3-O-methyltransferase
ASMT	N-acetylserotonin methyltransferase
IAA	3-Indoleacetic acid
GA	gibberellin
