# Supplementary material for: Overexpression of TaCOMT Improves Melatonin Production and Enhances Drought Tolerance in Transgenic Arabidopsis
Source: Int J Mol Sci. 2019 Feb 2;20(3):652. doi: 10.3390/ijms20030652 (PMC6387377; doi:10.3390/ijms20030652)
Supplement: Supplementary file 1 [file ijms-20-00652-s001.zip › supplementary files/Supplementary material--2019.1.25.docx]

**Overexpression of *TaCOMT* improves melatonin production and enhances drought tolerance in transgenic *Arabidopsis***

**Wen-Jing Yang^1,^ ^2†^,** **Yong-Tao Du^2†^, Yong-Bin Zhou^2^, Jun** **Chen^2^, Zhao-Shi Xu^2^, You-Zhi Ma^2^*, Ming Chen^2^* and Dong-Hong Min^1^***

**Supplementary information**

**Figure S1. The stress associated genes not response to drought in transgenic *Arabidopsis*.** The two-week-old Arabidopsis seedlings were placed on filter paper to simulate drought for two hours, and then the transcription level of drought-responsive genes was analyzed (A-C). Expression of *AtActin* as a loading control. An ANOVA test identified significant differences (*p < 0.05, **p < 0.01).

**Figure S2. The phenotype of TaCOMT transgenic lines under heat treatment.** The phenotype of WT and transgenic lines were treated with 44 for 4.5 h.

**Figure S3. Hypocotyl length under PAC treatment in *Arabidopsis*.** Phenotypes of WT and *TaCOMT* transgenic *Arabidopsis* seeds grown on medium containing 0, 1, 10, and 20 nΜ PAC for a week (A). Statistical results of hypocotyl length of WT and transgenic plants treated with different concentrations of PAC (B). At least 25 seedlings per phenotype were counted. The data are shown as the means ± SDs (n = 30) of three experiments. ANOVA test identified significant differences (*p < 0.05, **p < 0.01).

**Figure S4. The phenotype of early flowering in *TaCOMT* transgenic lines.** Three-week-old seedlings under normal conditions. After six days, *TaCOMT*-overexpression lines showed an earlier flowering phenotype than WT (A). The number of rosette leaves in flowering of transgenic and WT lines (B). The leaves of three-week-old *Arabidopsis* *thaliana* seedlings were used to analyze the expression of flowering-associated genes (C). Expression of *AtActin* was used as a loading control. At least 25 seedlings per phenotype were counted. The data are shown as the means ± SDs (n = 25) of three experiments. ANOVA test demonstrated significant differences (*p < 0.05, **p < 0.01).

**Table S1. Primers used for qRT-PCR assays.**
