# Supplementary material for: Overexpression of TaCOMT Improves Melatonin Production and Enhances Drought Tolerance in Transgenic Arabidopsis
Source: Int J Mol Sci. 2019 Feb 2;20(3):652. doi: 10.3390/ijms20030652 (PMC6387377; doi:10.3390/ijms20030652)
Supplement: Supplementary file 1 [file ijms-20-00652-s001.zip › supplementary files/Table S1--2018.12.9.docx]

|  | Forward Primer | Reverse Primer |
| --- | --- | --- |
| RT-TaCOMT | AAGGCAAGGTGGTAGTCGT | CCTGCGTAGATGTAGGTGGTC |
| RT-TaActin | CTCCCTCACAACAACCGC | TACCAGGAACTTCCATACCAAC |
| RT-AtActin | CTGGAATGGTGAAGGCTGGTT | CGATTGGATACTTCAGAGTGAGGAT |
| RT-AtRAB18 | TAGCTCGGAGGATGATGGACA | CCGTAGCCACCAGCATCATA |
| RT-AtRD29A | TGCACCGGCTCATTCTGTAA | GCAGAGAGACCGGAGTGTTC |
| RT-AtKIN2 | AACAAGAATGCCTTCCAAGC | TTAACACCTCCCACTGCCG |
| RT-AtDREB2A | GGTTGGCCCAATGATGTGGA | CCATCCTTTCCCTCGAGCTG |
| RT-AtKIN1 | CTGGAGCTGGAGCACAACAG | TTGTTCAGGCCGGTCTTGTC |
| RT-AtP5CS1 | TGGGGGTAAACTCATTGGCTT | TGCGCTTTGCCATATCCGTA |
| RT-AtCOR47 | CGGTCGCAACAGAGGAATCA | CTCGAGCGTCGTTGTCTCTT |
| RT-AtCOR15A | GCGATGTCTTTCTCAGGAGC | CGAACTGAGTTTTCTGGCCG |
| RT-AtABA2 | GGCGTTGGTCCACATTCT | TCCTCCGTTCTTTCTTCCTC |
| RT-AtABI3 | CCTGCTCCAAACTATCCGC | ACCTCCTCTGTCTCGCCATC |
| RT-AtABI1 | GTGGCTGGAGAAGTGGAAGA | GACGAAGATGTGAGACGGGA |
| RT-AtNCED3 | ATTGCTTCTGCTTCCATCTCT | TTCTGTTGACCATCCCTGCT |
| RT-AtCYP707A3 | GGCGGCTCTGTTTCTCTGT | ATCCGTATCTTCTCTGTTTTGC |
| RT-AtHAB1 | ACTGCTGTTGTTGCCTTGG | TCATCCTCTCTATCTGGTTTGTG |
| RT-AtGA20OX1 | TTAGGCGTAAAACGGGACT | CTTGGTGAAGGATGGTAAGAGA |
| RT-AtGA2OX1 | CCAATGGTGGTAAGAATGTGA | CAATGAAGGTCCAGCGAAGTA |
| RT-AtGA3OX1 | ACAAGTGGACCCCTAAAGACG | TTGGACAGGTAGCCCGAA |
| RT-AtFT | CTTGGCAGGCAAACAGTGT | CTAAAGTCTTCTTCCTCCGCAG |
| RT-AtSOC1 | AGCTCTCTGAAAAGTGGGGAT | TTGAAGAACAAGGTAACCCAATG |
