# Supplementary figures and images for: Overexpression of TaCOMT Improves Melatonin Production and Enhances Drought Tolerance in Transgenic Arabidopsis
Source: Int J Mol Sci. 2019 Feb 2;20(3):652. doi: 10.3390/ijms20030652 (PMC6387377; doi:10.3390/ijms20030652)

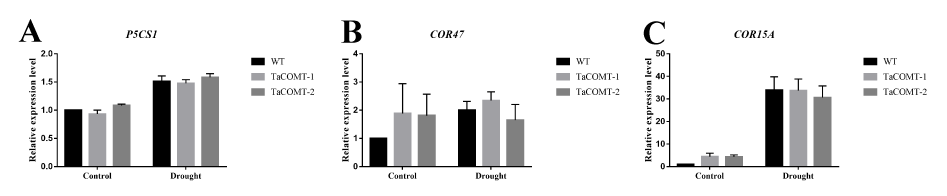

Supplement: Supplementary file 1 [file ijms-20-00652-s001.zip › supplementary files/Figure S1.tif]

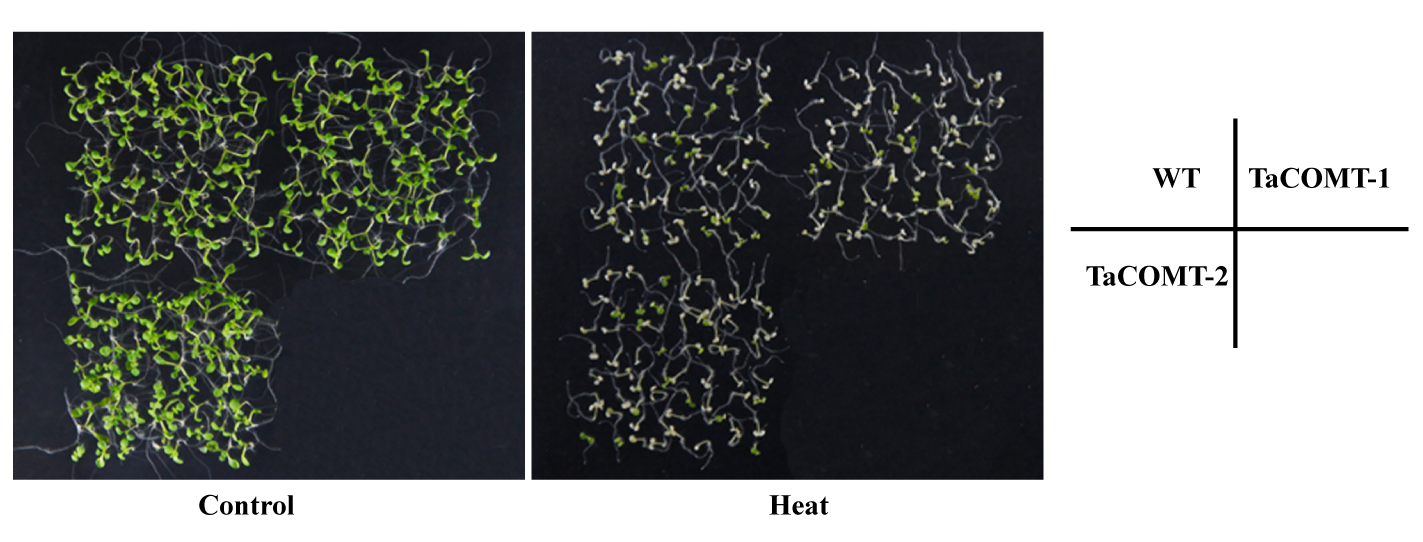

Supplement: Supplementary file 1 [file ijms-20-00652-s001.zip › supplementary files/Figure S2.tif]

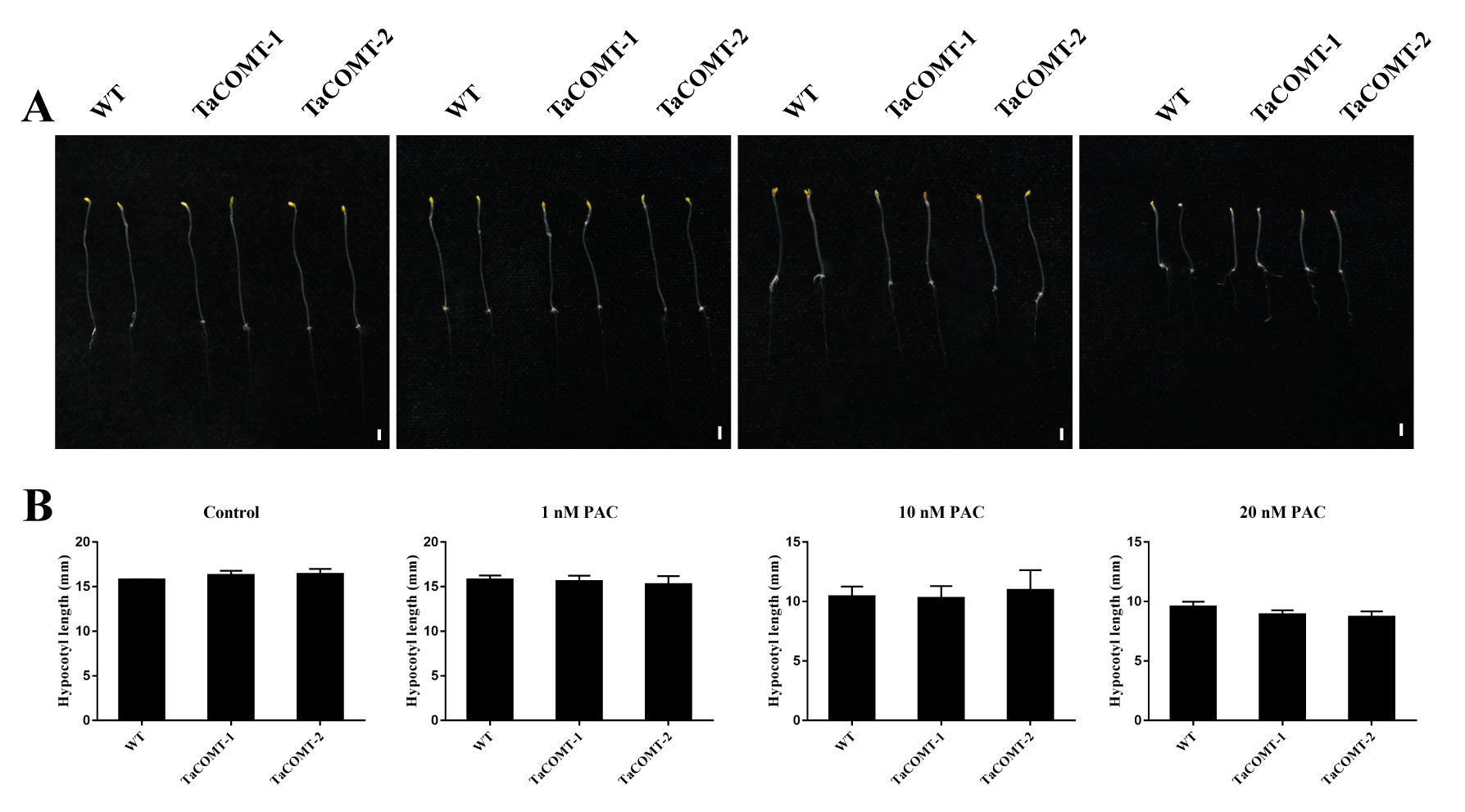

Supplement: Supplementary file 1 [file ijms-20-00652-s001.zip › supplementary files/Figure S3.tif]

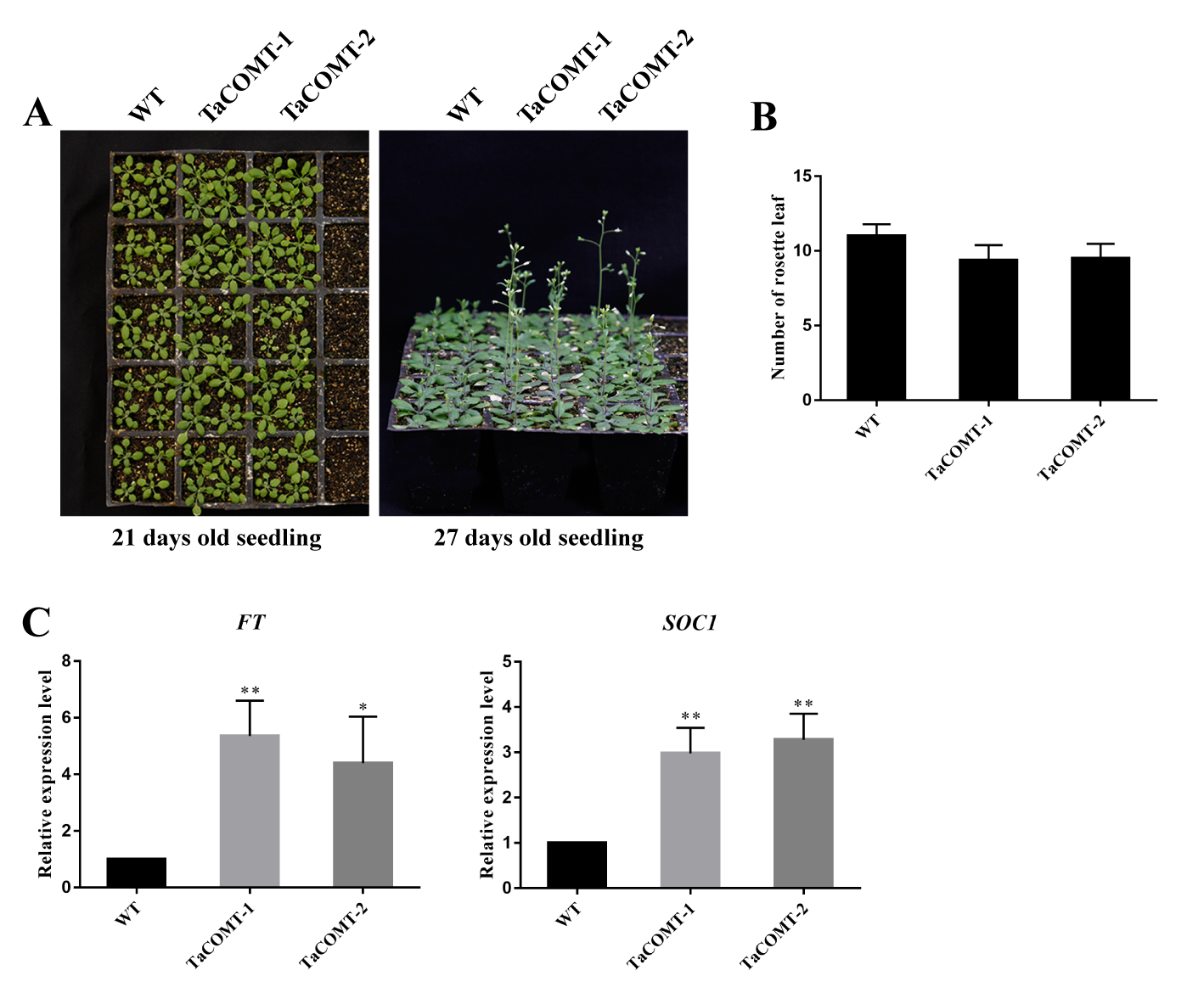

Supplement: Supplementary file 1 [file ijms-20-00652-s001.zip › supplementary files/Figure S4.tif]
